# Optimizing Performance Nutrition for Adolescent Athletes: A Review of Dietary Needs, Risks, and Practical Strategies

**DOI:** 10.3390/nu17172792

**Published:** 2025-08-28

**Authors:** Sotiria Everett

**Affiliations:** Department of Family Medicine, HSC L-3, 086, Renaissance School of Medicine, Stony Brook University, Stony Brook, New York, NY 11794-8036, USA; sotiria.everett@stonybrookmedicine.edu

**Keywords:** adolescent athletes, sports nutrition, energy availability, macronutrient requirements, hydration strategies, micronutrient deficiencies, relative energy deficiency in sport (RED-S), disordered eating, ergogenic aids, nutrition education

## Abstract

Adolescent athletes face unique nutritional challenges due to the simultaneous demands of growth, development, and athletic performance. This review synthesizes current evidence on energy and macronutrient requirements, hydration strategies, and key micronutrients, including iron, calcium, and vitamin D, which are essential for supporting health and performance in youth sport. It explores the physiological risks associated with low energy availability (LEA), while emphasizing the importance of carbohydrate and protein timing, quality, and distribution. The review also evaluates the role of dietary supplements and ergogenic aids, including creatine and energy drinks, highlighting safety concerns and advocating for a food-first approach. Practical strategies for nutrition education, interdisciplinary collaboration, and individualized care are presented to guide healthcare professionals, coaches, and caregivers in fostering sustainable, performance-supportive eating habits. By aligning intake with training demands and developmental needs, adolescent athletes can optimize performance, recovery, and long-term well-being.

## 1. Introduction

Adolescence is a critical period of rapid physical, cognitive, and emotional development [1]. For young athletes, these changes are compounded by the physiological demands of regular training and competition, resulting in elevated and highly individualized nutritional needs. Proper nutrition during this stage is essential not only for supporting healthy growth and maturation but also for enhancing athletic performance, recovery, and long-term health outcomes. This review explores the unique nutritional needs of adolescent athletes and outlines practical strategies to support optimal health, performance, and long-term well-being.

Implementing individualized nutrition strategies that meet energy, macronutrient, and micronutrient needs is essential not only for supporting healthy development and athletic performance but also for promoting sustained participation and lifelong health.

Adolescence is also a formative time for establishing dietary habits and body image perceptions. Young athletes may be particularly susceptible to external influences, such as those from parents, peers, and coaches, which can increase the risk of disordered eating behaviors [2]. Optimizing nutrition during this stage can help foster healthy, body-positive habits that athletes can carry into adulthood and, if applicable, into elite or professional level competition. Adequate nutrition also supports key physiological systems, including musculoskeletal growth, cardiorespiratory fitness, neurodevelopment, and immune function, all of which are critical for athletic potential and resilience.

For the purpose of this review, the term adolescent athletes refers to a period between childhood and adulthood and includes girls aged 12–18 years and boys aged 14–18 years (Tanner stages 3 and 4 of sexual maturation), who engage in structured physical training for more than 60 min per day, 5–6 days per week [3]. This level of activity typically includes skill development and conditioning aimed at competitive performance across school-based, club, or community sports programs.

Despite increasing interest in sports nutrition for youth, most existing guidelines are based on adult populations [4]. This gap is largely due to ethical and logistical challenges in conducting research with adolescents. While global estimates are not available, data from the United States indicates that approximately 60% of adolescents between the ages of 13–17 played organized sports in 2023 and that statistic is expected to rise [5,6], highlighting urgent need for quality studies that address the unique and individualized nutritional demands of adolescent athletes.

This review summarizes the findings from published research on sports nutrition for young athletes, specifically energy needs, nutrient needs, nutritional-related risks (e.g., low energy availability), and hydration. The role of ergogenic aids and dietary supplements is examined. The aim of this review is to provide practical guidance for healthcare providers working with such individuals.

## 2. Energy Requirements

Determining energy requirements in adolescent athletes is a complex and individualized process influenced by numerous physiological and training-related variables. While the question, “How many calories do growing athletes need?” may seem straightforward, the answer depends on factors such as age, gender, body composition, and the frequency, intensity, and duration of physical activity.

Adequate caloric intake is essential not only to support athletic performance but also to sustain normal growth and development. During adolescence, periods of rapid growth, such as peak height velocity (PHV), which typically occurs between ages 11.6–12.1 years in girls and 13.8–14.1 years in boys, [7,8] require sufficient energy to maintain a linear growth trajectory. Inadequate energy intake during this critical window can disrupt growth and maturation [9].

Despite the importance of meeting energy demands, there is no universally accepted gold standard for estimating energy needs in adolescent athletes. Direct measurement methods such as indirect calorimetry and doubly labeled water (DLW) provide accurate assessments of energy expenditure in individuals, including adolescent athletes, but are impractical due to cost, time, and equipment requirements. Traditional predictive equations (e.g., Harris-Benedict, Schofield, Cunningham) were developed for adults and may not be reliable measures of resting metabolic rate (RMR) in adolescents [10,11]. It has been estimated that the additional caloric cost of tissue synthesis in growing adolescents at approximately 2 kcal/g of daily weight gain, though this is difficult to quantify in practice [12]. While growth contributes to overall energy needs, the energy demands of physical activity are typically more substantial.

To address the limitations of traditional equations a predictive model based on data from 126 adolescent athletes (average age 16.5 years) has been developed [11].

RMR (kcal/day) = 11.1 × Body Mass (kg) + 8.4 × Height (cm) − (340 for males or 537 for females)

This equation may better estimate baseline energy needs for adolescent athletes in clinical and sports nutrition settings. Table 1 summarizes the key recommendations for energy, macronutrients, hydration and key micronutrients discussed in the sections below.

Beyond RMR, calorie needs should be based on the total daily energy expenditure (TEE) which applies a physical activity level (PAL) multiplier to RMR. This method is both practical and feasible in clinical and dietetic settings. While the World Health Organization [35] recommends PAL values of 2.0–2.4 (FAO/WHO) for vigorously active adults, these values may not be appropriate for adolescents. Research assessing physical activity levels among adolescent athletes aged 12–18 years found that the mean PAL in this population was significantly lower than adult-based recommendations [36]. As a result, a more appropriate PAL range of 1.75 to 2.05 was proposed based on this data. Using adolescent-specific PAL values provides a more accurate and developmentally appropriate framework for estimating energy requirements.

For effective sports nutrition guidance, it is essential to assess energy intake relative to energy expenditure, as low energy availability (LEA) and symptoms of relative energy deficiency in sport (RED-S) can develop in adolescent athletes engaged in high training volumes. LEA occurs when energy intake (EI) minus exercise energy expenditure (EEE), normalized to fat-free mass (FFM), is insufficient to support physiological health and athletic performance. While standardized thresholds for LEA have not been established all populations, an intake of <30 kcal/kg FFM/day has been identified as a risk threshold in female athletes, with ≥45 kcal/kg FFM/day considered optimal for maintaining physiological function [13,37]. Specific cut-off values of LEA in adolescent athletes have not been established, however a chronic energy deficit in developing athletes is associated with a range of adverse health outcomes, including delayed puberty, menstrual dysfunction, impaired bone health, short stature, disordered eating behaviors, and increased risk of injury [38].

In adolescent athletes, LEA disrupts the endocrine system in ways that can significantly impair recovery and increase injury risk. During adolescence, a critical period for growth and development, hormonal balance is essential for bone health, muscle repair, and overall physiological resilience. LEA is associated with elevated cortisol levels, which can impair immune function and increase protein breakdown, thereby delaying tissue repair and recovery. Simultaneously, reductions in insulin-like growth factor-1 (IGF-1), a key anabolic hormone, compromise bone formation and muscle regeneration, heightening susceptibility to stress fractures and soft tissue injuries [39].

Sex hormones such as estradiol and testosterone are also affected. In females, reduced estradiol levels due to LEA can lead to menstrual dysfunction and decreased bone mineral density, increasing the risk of overuse injuries. In males, suppressed testosterone levels impair muscle protein synthesis and recovery capacity. These hormonal disruptions are particularly concerning in adolescents, as they interfere with peak bone mass accrual and neuromuscular development, potentially leading to long-term consequences for health and athletic performance [40].

Another factor that may influence susceptibility to LEA in females is gynecological age, which represents the number of years since menarche. Research has demonstrated that adolescent females with a younger gynecological age are more prone to anovulation and shortened luteal phases. A study looking at a five-day period of reduced energy availability led to a significant decrease in luteinizing hormone (LH) pulse frequency in adolescents, but not in adults, emphasizing the heightened physiological sensitivity of adolescents to energy deficits [14]. Furthermore, LEA and low body weight in pubescent females have been linked to reduced bone mineral density and compromised bone microarchitecture, thereby increasing the risk of stress fractures [41].

While LEA and RED-S are more frequently described within female athletes, male athletes also experience negative metabolic and endocrine alterations due to energy imbalances. A recent longitudinal study among male high school elite athletes found RED-S symptoms, including low bone mineral density with suboptimal bone accrual during a critical period [42]. In this study, mean energy availability remained stable at 50 ± 16 kcal/kg FFM/day; however, the authors note that periodic measurements of energy availability may have been insufficient, and more research is needed to determine LEA thresholds male adolescents.

Recent research consistently demonstrates that adolescent athletes across various sports often fail to meet their energy requirements, highlighting the need for improved nutritional assessment and monitoring. For instance, Lehmann et al. reported that adolescent rugby players frequently exhibited suboptimal energy availability, with mean values ranging from 38.5 to 47 kcal/kg fat-free mass/day depending on training intensity [43]. Similarly, Bell et al. found that although adolescent volleyball players consumed more calories than non-athletes, they only met 60% of their estimated energy requirements, compared to 74% in non-athletes, indicating a significant energy deficit [44]. In Portuguese adolescent male soccer players, Martinho et al. observed a mean energy intake of 1929 kcal/day against a predicted expenditure of 3568 kcal/day, resulting in a substantial daily deficit of 1639 kcal [45]. This pattern of under-fueling is echoed youth tennis players, where average daily energy intake ranged from 2027 kcal/day in under 12 s to 2275 kcal/day in under 14 s and 2243 kcal/day in those aged 16 years and older, with no significant differences across age groups despite increasing energy demands [46]. These deficits are particularly concerning given the high training volumes and match loads of tennis, which often exceed three hours per day and can result in energy expenditures of 650–1000 kcal per match [46]. Additionally, energy demands extend beyond the training window. Inadequate compensation for these elevated demands may impair growth, delay recovery, and increase the risk of injury and long-term health complications associated with low energy availability and RED-S [42]. These findings emphasize the importance of assessing energy balance and ensuring adequate intake to support both performance and development in adolescent athletes.

## 3. Carbohydrates

Carbohydrates (CHO) are the primary fuel source for high-intensity and endurance exercise and play a critical role in supporting athletic performance, recovery, and overall health in adolescent athletes [47]. Despite their importance, research suggests that carbohydrate intake is often insufficient among youth athletes, particularly those engaged in aerobic or team-based sports [47,48].

Adolescents may have a reduced capacity to store glycogen compared to adults and may rely more heavily on exogenous carbohydrate sources during prolonged or intense activity [15]. Glycogen depletion can contribute to fatigue in young athletes, making it essential to replenish glycogen stores between training sessions and competitive events, especially during tournaments or multi-day competitions.

Daily carbohydrate recommendations for young athletes range from 6–10 g/kg per day, depending on exercise intensity [49]. Food sources to meet carbohydrate needs include whole grains (e.g., bread, pasta, rice, oats), starchy vegetables (e.g., potatoes, squash), fruits, legumes, low-sugar cereals, dairy products like yogurt, and sports nutrition products such as drinks, gels, and bars.

Carbohydrate timing is a critical component of nutritional strategies aimed at optimizing athletic performance. Pre-exercise carbohydrate consumption has been shown to enhance performance by increasing muscle glycogen availability [50]. During prolonged exercise lasting longer than 60 min, the ingestion of 30–60 g of carbohydrates per hour—preferably in the form of rapidly oxidized sources such as glucose-fructose mixtures—can significantly improve endurance capacity [51]. Sports drinks containing approximately 6% carbohydrate concentration not only supply energy but also aid in maintaining hydration during activity. For post-exercise recovery, the timely intake of high-glycemic index carbohydrates within two hours is recommended to maximize glycogen resynthesis. Optimal replenishment of liver and muscle glycogen stores occurs with carbohydrate ingestion at a rate of 1.0–1.2 g/kg after competition or training and continuation of every two hours for the next 4–6 h post exercise [16,50,52,53].

Emerging evidence underscores the physiological and performance-related consequences of inadequate carbohydrate availability. In a controlled study of 18 adolescent male soccer players (mean age 16.2 ± 0.4 years), Stables et al. found that training with 0 g/kg carbohydrate intake led to a 28% increase in serum CTX (C-terminal telopeptide of type I collagen), a marker of bone resorption, compared to those consuming 1.5 g/kg carbohydrate (*p* = 0.03) [16]. Complementing these findings, Lodge et al. highlighted that low carbohydrate availability (LCA) may exert independent deleterious effects on bone metabolism, immune function, and hormonal regulation, even in the presence of adequate energy availability [17]. In female endurance athletes, LCA has been linked to increased injury risk, impaired menstrual function, and reduced endurance performance. These outcomes are particularly concerning given the high prevalence of suboptimal carbohydrate intake among female athletes and the underrepresentation of women in sports nutrition research. Together, these findings emphasize the necessity of aligning carbohydrate intake with training demands to mitigate the risks associated with low energy availability (LEA) and LCA, and to support both health and performance.

## 4. Protein

Protein is essential for supporting muscle protein synthesis (MPS), tissue repair, and overall growth in adolescent athletes. Current dietary guidelines for children and adolescents aged 8–19 years recommend a daily protein intake of 0.75–1.05 g/kg body weight [54]. However, within the sports nutrition literature, athletes are generally advised to consume between 1.4–2.0 g/kg/day to support muscle maintenance, recovery, and adaptation to training [18,19].

Beyond total intake, the timing and distribution of protein are critical for maximizing MPS. Studies recommend consuming 20–40 g of high-quality protein per meal, spaced every 3–4 h throughout the day [18,19]. This approach supports sustained anabolic signaling and muscle recovery, especially during the 16–48 h window following resistance exercise. Evidence suggests that adolescents should consume high quality protein shortly after exercise, ideally within the first hour, and to continue intake at regular intervals, as part of meals and snacks every 2–4 h, to sustain recovery and support muscle development [55]. A commonly recommended dose for post-exercise protein intake is 0.25–0.30 g/kg of body weight, which has been shown to optimize muscle protein synthesis (MPS) in both adults and adolescents. In a study by Mazzulla et al., whole-body net protein balance plateaued at ~0.30 g/kg post-exercise in adolescents aged 14–18 years (*n* = 13), indicating no additional benefit from higher doses. This supports the recommendation of ~20 g of high-quality protein per meal for a 70 kg athlete [20]. This amount can typically be met through whole foods, such Greek yogurt with fruit and granola, 1 cup of cottage cheese and pineapple, 16 oz of chocolate milk, or 2 tbsp peanut butter on whole grain bread with 8 ounces of soymilk. For example: A 70 kg male soccer player would benefit from approximately 18–21 g of protein in a recovery meal. A 60 kg female basketball player would require about 15–18 g. These amounts align with dose–response studies showing that ~20 g of protein per meal is sufficient to maximize MPS in most athletes.

Protein quality is equally important. The anabolic stimulation of MPS depends on the presence of essential amino acids (EAAs), particularly leucine. Of the 20 amino acids used in protein synthesis, nine are essential and must be obtained through the diet. Complete protein sources—such as eggs, dairy, lean meats, poultry, fish, and soy—provide all nine EAAs in optimal ratios. Plant-based proteins, including legumes, tofu, quinoa, and nuts, can support performance when consumed in complementary combinations (e.g., rice and beans) to ensure adequate EAA intake. This strategy is especially relevant for vegetarian and vegan athletes, who may require more total protein to match the EAA and leucine content of animal-based sources.

In addition to the inclusion of essential amino acids, sufficient energy intake is critical for optimal protein utilization to support muscle protein synthesis. In a study of young adult men undergoing a 40% energy deficit, muscle protein synthesis was significantly reduced, even with elevated protein intake, while resistance exercise helped mitigate this decline [56]. Although similar research in adolescents is lacking, their heightened physiological sensitivity during growth and development suggests that meeting energy requirements should be a priority to support protein utilization for both growth and athletic performance.

Leucine, a branched-chain amino acid, is a key regulator of MPS through the activation of the mTORC1 pathway. In adult populations, ~3 g of leucine per meal is recommended to maximize anabolic responses, typically achieved through ~20–25 g of high-quality protein [55]. While direct evidence in adolescents is not available, leucine-rich meals are likely beneficial during growth and training. Dietary sources include chicken breast (~2.5 g leucine per 100 g), eggs (~1.2 g per 2 large eggs), and tofu (~1.4 g per 100 g).

A food-first approach is widely recommended for adolescent athletes to ensure a balanced intake of protein while minimizing reliance on supplements. This strategy not only supports optimal health and performance but also aligns with growing concerns about the environmental impact of diets high in animal-based proteins. In recent years, plant-based dietary patterns, including lacto-ovo-vegetarian and vegan diets, have gained popularity, particularly among younger generations. Surveys conducted among athletes in the UK and Europe report that approximately one-third of respondents either follow or are considering adopting plant-based diets [57]. Despite concerns about the adequacy of plant-based diets for meeting the protein needs of adolescent athletes, emerging evidence suggests that well-planned vegetarian and vegan diets can support both performance and nutritional adequacy. For example, a cross-sectional study involving 91 adolescent athletes in Brazil found that most participants met the American Dietetic Association’s recommended protein intake (1.2–2.0 g/kg/day), even among those with the lowest dietary carbon footprints [58]. The median intake of plant-based protein was 0.34 g/kg/day, while animal-based protein intake was 1.31 g/kg/day, indicating that environmentally sustainable diets can be compatible with athletic performance. These findings suggest that sustainable dietary patterns can be compatible with athletic performance and nutritional adequacy.

Additionally, consuming 30–40 g of casein protein before bedtime, found in foods like Greek yogurt or cottage cheese, has been shown to support overnight muscle repair and improve recovery, particularly in athletes undergoing intense training [19,59]. Despite generally adequate total protein intake among adolescent athletes, often exceeding 2.0 g/kg/day, observational data from NCAA Division I soccer players (*n* = 24) revealed that 68% of daily protein was consumed at dinner, with breakfast and lunch contributing only 14% and 18%, respectively [60]. This uneven distribution may impair MPS and recovery, emphasizing the need for balanced intake across meals. Improving the timing and distribution of protein consumption may further enhance performance, recovery, and long-term muscle development.

## 5. Dietary Fats

Dietary fats are essential for adolescent athletes, playing a critical role in supporting growth, maturation, and overall health. Fats provide a dense source of energy, aid in the absorption of fat-soluble vitamins (A, D, E, and K), and supply essential fatty acids necessary for hormone production and cellular function. Intake of fat by athletes should follow the public health guidelines of not exceeding 20–35% of total daily caloric intake [21,61]. For example, in a 2200-calorie diet, this equates to roughly 60–85 g of fat per day. Saturated fats should be limited to no more than 10% of total energy intake, i.e., approximately 24 g per day in a 2200-calorie diet [21]. Trans fats should be avoided as they have been shown to increase low density lipoprotein cholesterol (LDL-C) and reduce high density lipoprotein cholesterol (HDL-C). Foods that contain trans fats include fried foods, ultra-processed foods, shortening, and foods that contain shortening such as baked goods, pastries and donuts [23]. Preferred dietary fat sources include mono- and polyunsaturated fats such as those found in nuts, seeds (e.g., sunflower, pumpkin, flax, chia), avocados, olive oil, fatty fish (e.g., salmon, tuna), and nut butter. As part of a high quality diet for athletes, such fats can support long term cardiovascular health and serve as a valuable energy source, particularly during long-duration, low-intensity endurance exercise [23].

Evidence suggests that maximal fat oxidation rates (relative to lean mass) are slightly higher in athletes under 18 years of age [62]. This may be due to their limited glycogen storage capacity, making fat a more prominent fuel source during prolonged activity. Ensuring adequate fat intake is therefore important for sustaining energy levels and optimizing performance in young athletes.

Restricting fat intake, particularly in weight-sensitive or aesthetic sports such as gymnastics, figure skating, wrestling, and swimming, consumption may also contribute to LEA, which can impair growth, delay puberty, and increase the risk of injury and illness [63]. Furthermore, dietary fat restriction can increase the risk of essential fatty acid deficiencies and fat-soluble vitamin insufficiencies, including vitamin D. Regular nutritional monitoring is recommended for athletes in these sports to ensure balanced intake and long-term health.

Omega-3 polyunsaturated fatty acids (PUFAs), particularly docosahexaenoic acid (DHA), have been associated with anti-inflammatory effects, improved recovery, and potential benefits for sleep and brain health [64]. In adolescent athletes, DHA is of particular interest due to its role as a structural component of neuronal membranes and its involvement in neurogenesis, synaptic function, and cognitive processes such as memory and learning. A randomized controlled trial in adolescent athletes (14–18 years) found that DHA supplementation (2 g/day) following sport-related concussion resulted in earlier symptom resolution and return-to-play clearance, although results were not statistically significant [65]. Complementing these findings, Lust et al. [66] reviewed experimental and early clinical evidence suggesting that DHA and eicosapentaenoic acid (EPA) may attenuate neural damage associated with sports-related concussions (SRC) and sub concussive impacts (SCI). DHA supplementation in collegiate football players was associated with reduced levels of neurofilament light (NF-L), a biomarker of neural injury, indicating a potential neuroprotective effect [67]. While DHA appears more effective in preserving structural brain integrity, EPA may offer additional benefits for mood regulation, including reductions in depression-like symptoms, which are common in athletes with a history of SRC. These findings highlight the potential clinical relevance of omega-3 s in supporting recovery and neurological health in youth athletes, though further research is needed to establish optimal dosing strategies for this population.

Omega-3 fatty acids have also been associated with reduced inflammation and accelerated recovery from exercise induced muscle damage (EMID), though evidenced from studies remains mixed. Although not specific to adolescents, a systematic review of 13 RCTs found that EPA/DHA supplementation reduced post-exercise inflammation and muscle damage, but showed limited impact on performance outcomes [68]. A recent meta-analysis found that omega-3 supplementation (0.8–3 g/day) lowered inflammatory markers (IL-6, TNF-ɑ, and CRP levels) following exercise-induced muscle damage, particularly with longer durations and combined aerobic–anaerobic protocols [69]. Evidence from collegiate athletes indicates that dietary omega-3 intake is inadequate. A large study of NCAA Division I athletes revealed suboptimal omega-3 status, with an average Omega-3 Index of 4.3%, below the optimal target of ≥8%, and only 6% meeting intake recommendations, highlighting the need for athlete-specific guidelines and interventions [70].

## 6. Hydration

Hydration is a critical component of performance, recovery, and safety in adolescent athletes. Due to their unique physiology, including a higher surface area-to-body mass ratio and less efficient thermoregulation, children and adolescents are particularly vulnerable to dehydration and heat-related illnesses. Even a 1–2% reduction in body mass due to fluid loss can impair aerobic performance and cognitive function [24].

Hydration recommendations for athletes should be tailored based on sweat rate, and hydration education is important for young and adolescent athletes [25]. Sweat rate can vary significantly depending on exercise intensity, environmental conditions, and individual physiology.

Athletes should begin exercise well-hydrated and follow a structured hydration strategy to minimize sweat-induced fluid deficits. During activity, young adolescents should aim to consume approximately 3–8 oz (~90–240 mL) of fluid every 20 min, while older adolescents may require up to 1.0 L per hour, depending on sweat rate and environmental conditions [26]. Monitoring body weight before and after exercise is a practical method to estimate fluid losses, with each pound lost equating to approximately 16–20 ounces (~480–600 mL) of fluid that should be replaced [26]. Adolescents have a reduced ability to dissipate heat through sweating and are more susceptible to heat stress [71]. Sodium replacement is also important, particularly for athletes with high sweat rates, to support fluid retention and prevent muscle cramping. During prolonged or intense activity, especially in hot environments, sports drinks can be beneficial. These drinks typically contain 6–8% carbohydrates, 185–160 mg of sodium, and 35–90 mg of potassium per 8 oz (240 mL) serving. Sodium is the primary electrolyte lost in sweat and plays a key role in fluid retention and rehydration. In hot or humid environments, fluid needs increase due to greater sweat losses.

Many commercially available sports drinks contain artificial food colorings (AFCs), such as Red #40, Yellow #5, Yellow #6, and Blue #1. While these additives enhance visual appeal, emerging research has raised concerns about their potential health effects in children and adolescents. Some studies suggest that excessive intake of AFCs may be associated with behavioral changes, including attention-related symptoms, and may carry carcinogenic or neurotoxic risks [72]. Although specific research on the effects of AFCs in sports drinks consumed by adolescent athletes is limited, caution is warranted. Parents and athletes concerned about AFC exposure should consider selecting sports drinks that are free from artificial dyes or opt for naturally colored or clear alternatives.

## 7. Micronutrient Considerations

### 7.1. Iron

Iron is a vital micronutrient for adolescent athletes due to its role in oxygen transport, energy metabolism, and growth. During adolescence, iron requirements increase to support rapid growth and expanding blood volume. Iron deficiency is particularly prevalent in the athletic population, particularly adolescents, affecting approximately 20% of athletes overall and more than 35% of female athletes, and up to 52% of female adolescent athletes [73,74]. Female athletes and those participating in endurance sports are at heightened risk due to factors such as menstrual blood loss, lower dietary iron intake, and increased iron turnover from high training loads [75].

A contributing factor of iron deficiency in sport is reduced iron absorption following exercise, driven by elevated levels of hepcidin, the master regulator of iron homeostasis. Hepcidin inhibits iron efflux from enterocytes and macrophages, thereby reducing dietary iron absorption and iron recycling. Its expression increases in response to inflammation and elevated iron levels.

Exercise-induced inflammation causes a transient rise in hepcidin, temporarily impairing iron absorption. However, recent studies suggest that chronic training may suppress hepcidin expression, especially in iron-deficient athletes. For example, Peeling et al. [27] found that post-exercise hepcidin levels were elevated only in athletes with moderate to high baseline ferritin, while those with low iron stores showed a blunted hepcidin response. Similarly, Kobayashi et al. reported that continuous training in female high school dancers decreased hepcidin levels compared to rest periods, likely due to increased erythropoietin and hemolysis [28]. Notably, serum 25(OH)D (vitamin D) levels were positively correlated with iron markers, suggesting a potential modulatory role of vitamin D in iron metabolism.

Iron depletion, even in the absence of anemia, can impair aerobic capacity, cognitive function, and immune health [76]. The Dietary Reference Intakes (DRIs) for iron are 11 mg/day for males and 15 mg/day for females aged 13–18 years [29]. Iron losses may occur through sweat, urine, feces, and menstruation. Vegetarian or vegan diets may increase risk of iron deficiency if not properly planned.

Given the high prevalence of iron deficiency among adolescent athletes, particularly during a critical period of growth, development, and skill acquisition, regular screening is recommended, especially in individuals presenting with symptoms such as fatigue, reduced endurance, or frequent illness. Assessment should include serum ferritin levels, which serve as a reliable indicator of iron stores. In healthy male and female athletes over the age of 15 years, ferritin concentrations below 15 µg/L are considered indicative of depleted iron stores, while values between 15–30 µg/L suggest low iron reserves (Table 2). Therefore, a clinical cut-off of 30 µg/L is appropriate for this age group. For younger populations, recommended ferritin thresholds are 15 µg/L for children aged 6–12 years and 20 µg/L for adolescents aged 12–15 years [77].

Oral iron supplementation is the standard treatment for iron deficiency, typically at doses of 60–120 mg elemental iron. To maximize absorption, iron should be taken when hepcidin levels are low, ideally in the morning, away from meals, and with a source of vitamin C. A recent study in iron-deficient women demonstrated that taking iron with orange juice significantly improved absorption, while co-ingestion with coffee or breakfast reduced absorption by 54–66%, even when vitamin C was present [78]. Iron absorption was also lower in the afternoon, likely due to elevated hepcidin levels.

Formulation and tolerability are important considerations. Ferrous salts, such as ferrous sulfate, ferrous fumarate, and ferrous gluconate are effective but may cause gastrointestinal side effects. Ferric iron complexes (e.g., ferric polysaccharide, polymaltose, polydextrose) are generally better tolerated but have lower efficacy and limited data in athletic populations. While vitamin C enhances iron absorption, co-supplementation with ferrous salts may increase oxidative stress in the gastrointestinal tract, potentially contributing to inflammation [79].

In cases of severe iron deficiency anemia or when oral iron is contraindicated, intravenous iron therapy may be considered. This approach bypasses gastrointestinal absorption and can rapidly restore iron levels, but it is costly and should be administered under medical supervision [80].

Dietary sources of iron include lean meats, eggs, leafy green vegetables, fortified cereals, and legumes. In cases of confirmed deficiency, healthcare professionals should monitor iron status and implement dietary interventions or supplementation as needed to restore optimal levels and support both health and athletic performance.

### 7.2. Calcium and Vitamin D

Calcium and vitamin D are essential for bone health, particularly during adolescence when peak bone mass is being established. Calcium needs are highest during the pubertal growth spurt, with a recommended intake of 1300 mg/day for both males and females aged 14–18 years [30]. Despite this, many adolescents fail to meet these requirements, increasing the risk of suboptimal bone development and future skeletal issues [31]. Adolescent athletes should aim to include dietary sources of calcium in most meals and snacks consumed during the day to meet their requirements. Food sources high in calcium include yogurt (488 mg in 8 ounces), milk (305 mg in 1 cup), soymilk (300 mg in 8 ounces), and cooked leafy greens such as spinach (245 mg in 1 cup). Fortified plant-based milks and juices may also be sources of calcium for athletes who do not tolerate dairy.

Vitamin D plays a critical role in calcium absorption, immune regulation, and muscular function—key components of athletic performance and recovery. Deficiency has been linked to muscle weakness, inflammation, and delayed recovery [81]. While research specific to adolescent athletes remains limited, studies in young athletic populations offer valuable insights. A 2024 study of elite soccer players (mean age 22.3 ± 2.1 years, *n* = 40) found that athletes with serum 25(OH)D levels ≥ 30 ng/mL demonstrated a 9.8% improvement in change of direction (COD) performance compared to those with lower levels (*p* < 0.01) [82]. While not conducted in adolescents, these findings suggest a potential role for vitamin D in neuromuscular adaptation relevant to agility. Similarly, in a cross-sectional study of 125 physically active children aged 10–14 years, Bezrati et al. found that each 10 ng/mL increase in plasma 25(OH)D was associated with a 5.2 cm increase in vertical jump height and a 7.4% improvement in handgrip strength (*p* < 0.05), independent of age, maturity, and body composition [32].

In addition to performance, vitamin D status may influence injury risk. Although not focused on adolescents, a 2021 narrative review identified vitamin D deficiency as a significant risk factor for stress fractures in athletes, particularly when serum levels fall below 30 ng/mL [33]. These findings can inform adolescent care, especially given that adolescents are at risk for vitamin D inadequacy due to limited sun exposure, darker skin pigmentation, indoor training, and sunscreen use. NHANES data (2011–2014) indicate that 22.7% of U.S. adolescents aged 12–19 are at risk of inadequacy, and 4.8% at risk of deficiency [34]. Given the prevalence and implications of deficiency, screening and supplementation are recommended for at-risk athletes [83]. The recommended dietary allowance (RDA) for vitamin D ranges from 600–800 IU/day (15–20 µg), though supplementation may be necessary due to limited dietary sources such as fortified milk, eggs, and fatty fish [30]. Monitoring serum 25(OH)D levels is advised to ensure optimal status.

## 8. Ergogenic Aids and Dietary Supplements

Among adolescent athletes, the use of dietary supplements is increasingly common, often driven by goals related to strength development, recovery, body composition, and competitive performance [84]. Commonly used supplements include protein, creatine, and stimulants such as caffeine (often consumed through energy drinks). While some of these aids may offer benefits when used appropriately, it is essential that athletes, caregivers, and coaches understand their mechanisms, efficacy, safety, and legality. Evidence-based guidance is critical to ensure that supplement use support rather than compromises health and performance.

The rise of social media, influencer marketing, and peer influence has significantly shaped adolescents’ perceptions of supplements. Young athletes are frequently exposed to promotional content that may exaggerate benefits or downplay risks, leading to the misconception that supplements are necessary for achieving athletic success or ideal body image. Without proper education, this exposure can contribute to unsafe or unnecessary supplement use.

Healthcare professionals play a vital role in providing accurate, age-appropriate information and helping adolescent athletes critically evaluate supplement claims. This includes addressing common concerns, such as the safety of protein powders, and promoting a food-first approach that prioritizes whole foods to meet nutritional needs before considering supplementation. Table 3 provides a summary of ergogenic aid and supplement recommendations discussed in this review.

### 8.1. Creatine Supplementation in Adolescent Athletes

Creatine monohydrate is one of the most extensively researched and effective dietary supplements for enhancing high-intensity exercise performance, increasing intramuscular phosphocreatine (PCr) stores, and supporting training adaptations. It is naturally found in protein-rich foods such as meat and fish, with typical dietary intake ranging from 1–2 g per day. Endogenously, creatine is synthesized from the amino acids methionine, glycine, and arginine, and approximately 1–2% of intramuscular creatine is metabolized daily into creatinine.

Although research on creatine use in adolescent athletes is limited, emerging evidence suggests that it may be both safe and beneficial when used under appropriate supervision. A review highlights that creatine supplementation, when administered within recommended dosages and under professional guidance, may offer a favorable safety profile for youth athletes engaged in serious or competitive training [88]. For example, a 2017 study involving elite youth soccer players (mean age 17.0 ± 0.5 years) reported significant improvements in power output following a low-dose creatine regimen (0.03 g/kg/day) over seven days [89].

Surveys indicate that creatine is among the more commonly used supplements by adolescent athletes. One study found that 8.8% of boys and 1.8% of girls in grades 6–12 reported creatine use, with prevalence increasing substantially in upper high school grades [90]. Similarly, it was reported that 21% in boys and 3% in girls aged 13–18 years use creatine as an athletic supplement [91]. Despite its popularity, many adolescents may lack consistent or informed usage patterns, underscoring the need for education and oversight.

A recent position stand from the International Society of Sports on the safety and efficacy of creatine supplementation included guidelines for creatine among adolescents and suggests that creatine supplementation may be considered for adolescent athletes who [85]:

- Are engaged in structured, competitive training programs;
- Consume a well-balanced, performance-supportive diet;
- Demonstrate an understanding of appropriate creatine use;
- Adhere strictly to recommended dosages.

### 8.2. Energy Drinks and Energy Shots

Energy drinks (EDs) and energy shots (ESs) are widely consumed by adolescents and young adults, often marketed as performance-enhancing drinks. These products typically contain a combination of ingredients such as caffeine, taurine, guarana, ginseng, carnitine, B vitamins (B1, B2, B3, B5, B6, B9, B12), vitamin C, beta-carotene (vitamin A), vitamin D, electrolytes (e.g., sodium, potassium, magnesium, calcium), amino acids like tyrosine and L-theanine, and various sweeteners. The prevalence of each ingredient varies widely, with caffeine being the most consistently present and studied.

Caffeine is the primary ergogenic ingredient in most EDs and ESs, with doses around 200 mg (or >3 mg/kg body weight) shown to enhance aerobic performance, alertness, and reaction time [86]. While evidence supports caffeine’s performance-enhancing effects, these products also pose risks, including elevated blood pressure, sleep disturbances in adolescents, exacerbation of mental health conditions, physiological dependence, and potential for addiction. Moreover, many other ingredients in EDs and ESs remain poorly studied, both individually and in combination, raising concerns about the safety and efficacy of these multi-ingredient formulations, particularly in younger populations.

Adolescents represent a significant portion of ED consumers. A 2013 European survey reported that 68% of adolescents aged 10–18 years had tried EDs, compared to 30% of adults [87]. Similar trends are seen in the U.S., where EDs are frequently marketed toward active youth, including athletes. Despite growing concerns, regulatory oversight of ED marketing remains limited in both the U.S. and EU. This is concerning given the potential synergistic effects of caffeine (often ~200 mg per serving), taurine, guarana, and L-carnitine, which may increase the risk of adverse outcomes such as hypertension, tachycardia, hyperactivity, and sleep disturbances. Sports participation is often cited as a reason for ED use, and consumption has increased among adolescents and adults between 2003 and 2016 [92]. These trends underscore the need for further research and regulation to protect youth from the potential harms of EDs [92].

Health organizations, including the American Medical Association, recommend a maximum caffeine intake of 100 mg/day for adolescents and 500 mg/day for adults [93]. Given the potential for cumulative caffeine intake from multiple sources (e.g., coffee, tea, chocolate, pre-workout supplements), it is important for adolescent athletes to monitor total daily intake and avoid excessive consumption.

Due to the limited safety data and potential for adverse effects, energy drinks and energy shots are not recommended for children under 12, individuals who are pregnant or breastfeeding, or those with caffeine sensitivity. Adolescents aged 12–18 years should exercise caution and consult with a healthcare provider or guardian before consuming these products, especially in high doses (>400 mg/day).

### 8.3. Protein Supplements in Adolescent Athletes

Protein supplementation is a trending topic in general nutrition and has always been a hot topic in sports nutrition, as athletes of all ages strive to ensure proper amounts of protein for the attainment and maintenance of lean body mass. Protein supplements are widely used by athletes to support muscle recovery, enhance performance, and promote lean mass development. These products are available in various forms, including powders, bars, and ready-to-drink shakes, and are derived from a range of sources such as whey, casein, egg, beef, soy, and pea protein. They are further categorized by processing methods into concentrates, isolates, and hydrolysates, which differ in protein content and absorption rates.

Despite their popularity, most adolescent athletes can meet their protein needs through a balanced diet that includes whole food sources such as dairy, eggs, lean meats, legumes, and grains. Research indicates that adolescent athletes typically consume adequate protein, often 2–3 times the recommended protein levels, without the need for supplementation [94]. Therefore, protein powders should be viewed as a convenient option rather than a necessity, particularly useful in situations where whole food intake is limited, such as during travel, post-surgery, or between closely scheduled training sessions.

Adolescent athletes frequently use protein supplements to support muscle recovery, enhance performance, and prevent injury, often consuming them after training or competition. However, many young users lack adequate knowledge about the types of protein they consume, and the potential health risks associated with supplementation [95]. A growing concern involves the purchase of protein powders from unverified online sources, which may increase the risk of exposure to contaminants. Recent studies have highlighted the presence of heavy metals, including lead and cadmium, in some commercially available protein powders [96].

Given these risks, adolescent athletes should receive guidance from qualified professionals when considering supplementation. Emphasis should be placed on a food-first approach, with supplements used only when necessary and selected from reputable brands that undergo third-party testing.

### 8.4. Supplement Safety and a Food-First Philosophy

A food-first approach is strongly recommended for adolescent athletes, emphasizing that dietary supplements should never replace a well-balanced, nutrient-dense diet. Whole foods provide not only protein, carbohydrates, and fats but also essential micronutrients, fiber, and bioactive compounds that supplements cannot replicate. While supplements may offer convenience or targeted support in specific situations, such as during travel, injury recovery, or periods of high training load, they cannot compensate for poor dietary habits and are often costly.

When supplementation is deemed necessary, prioritizing product quality and safety is essential. Athletes should select supplements that have undergone third-party testing to ensure they are free from banned substances and accurately labeled. Reputable certification programs include NSF Certified for Sport, Informed Sport, and USP (United States Pharmacopeia). These quality assurance seals help verify that the product contains what it claims and is safe for use in sport, reducing the risk of inadvertent doping or exposure to harmful contaminants.

Educating athletes, coaches, and caregivers about supplement safety and regulatory oversight is critical to fostering informed decision-making and protecting the health and eligibility of young athletes.

## 9. Nutrition Education and Practical Strategies

Despite the growing demands of sport, research consistently shows that adolescent athletes often possess limited nutrition knowledge, which can lead to inadequate dietary intake, suboptimal fueling, and increased risk of nutrient deficiencies [97]. Potential outcomes of poor nutrition knowledge may lead to increased reliance on misinformation from peers, social media, or unqualified sources.

Recent studies highlight that adolescent athletes frequently obtain nutrition information from non-qualified or non-credible sources, which may contribute to misconceptions and suboptimal dietary practices. Bird and Rushton found that youth athletes often rely on coaches, teachers, peers, the internet, and social media for nutrition guidance, with only 16% reporting input from dietitians [98]. This reliance on informal channels was associated with poor understanding of dietary reference intakes and supplementation, underscoring the need for structured education led by qualified professionals.

Similarly, Deslippe et al. reported that adolescent athletes commonly seek nutrition advice from social media, peers, and coaches—sources that may lack formal nutrition training [99]. Although athletes were more likely than non-athletes to consult parents and coaches, these sources were not always validated. The study emphasized the importance of media literacy and critical evaluation skills to help adolescents navigate misinformation and unrealistic body ideals prevalent online.

Bourke et al. also found that 65% of New Zealand athletes used social media for nutrition information, with recreational athletes and females significantly more likely to do so than elite athletes [100]. While social media was valued for its accessibility and visual appeal, 84% of respondents expressed concern about the reliability of information. The authors stressed the need for nutrition professionals to engage with social media platforms to provide credible, practical, and engaging content, especially for athletes who lack regular access to professional guidance.

These gaps highlight the urgent need for targeted, evidence-based nutrition education that emphasizes the role of food in supporting growth, performance, recovery, and overall well-being.

Implementing a structured nutrition plan can be challenging for young athletes due to time constraints, academic responsibilities, social influences, and limited access to healthy food options. Therefore, an interdisciplinary approach is recommended—one that includes collaboration among healthcare providers, coaches, families, and qualified nutrition professionals. In the U.S., athletes and caregivers can locate a Registered Dietitian Nutritionist (RDN) with the Certified Specialist in Sports Dietetics (CSSD) credential through the Academy of Nutrition and Dietetics’ ‘Find a Nutrition Expert’ tool. International resources for locating qualified sports dietitians are listed in Table 4:

These resources can help connect athletes with professionals who understand the unique nutritional demands of adolescent sport.

Nutrition education should be developmentally appropriate, culturally sensitive, and focused on empowering athletes to make informed choices. Rather than emphasizing “good” vs. “bad” foods or rigid dietary rules, messaging should center on performance, energy availability, and recovery. Key topics should include:

- The importance of balanced meals and snacks throughout the day;
- Timing of nutrients before, during, and after training;
- Hydration strategies;
- Food-first approaches to meeting protein and micronutrient needs;
- Safe and informed use of supplements, when appropriate.

Additionally, body composition assessments should be approached with caution in this age group. Emphasizing performance, strength, and energy, instead of weight and aesthetics, can help prevent disordered eating and support a healthy body image. Adolescents should be taught autonomy in food choices and encouraged to view nutrition as a tool for achieving their athletic goals. Referrals to sports dietitians should be considered a standard part of care for adolescent athletes, particularly those with high training volumes, specialized dietary needs (e.g., food allergies, vegetarian/vegan diet patterns), or performance plateaus. With proper education and professional support, young athletes can develop lifelong habits that fuel both their sport and their health.

## 10. Conclusions

Adolescent athletes face unique nutritional demands due to the simultaneous pressures of growth, development, and athletic performance. This narrative review highlights the importance of meeting energy and macronutrient needs, maintaining hydration, and ensuring adequate intake of key micronutrients such as iron, calcium, and vitamin D. It also underscores the growing use of ergogenic aids and dietary supplements among youth athletes, emphasizing the need for evidence-based guidance and a food-first philosophy.

Nutrition education, delivered through interdisciplinary collaboration among healthcare providers, coaches, families, and registered dietitians, is essential to empower young athletes with the knowledge and tools to make informed dietary choices.

By prioritizing whole foods, aligning intake with training demands, and addressing barriers such as time constraints and social influences, adolescent athletes can optimize their performance, support healthy development, and establish lifelong habits that promote well-being both on and off the field.

## Figures and Tables

**Table 1:** Summary of energy, macronutrient, hydration and select micronutrient recommendations for adolescent athletes.

Nutrient	Recommended Intake/Target	Additional Considerations	Ref.
Energy	≥45 kcal/kg FFM/day (optimal) <30 kcal/kg FFM/day (risk for LEA)	RMR (kcal/day) = 11.1 × Body Mass (kg) + 8.4 × Height (cm)- 340 for males or 537 for females)	[11,13,14]
		PAL range: 1.75–2.05	
Carbohydrates	6–10 g/kg/day	Target based on training intensity	[15,16,17]
		Post-exercise 1.0–1.2 g/kg every 2 h for 4–6 h	
Protein	1.4–2.0 g/kg/day	Daily target: 20–40 g per meal every 3–4 h Post-exercise 0.25–0.30 g/kg	[18,19,20]
Fat	20–35% of total energy	Saturated fat <10%; trans fats avoided	[21,22,23]
Hydration	2.0–2.4 L/day baseline	During activity +3–8 oz (~90–240 mL) every 20 min	[24,25,26]
		Post-exercise fluid replacement 16–20 ounces (~480–600 mL) per pound lost	
Iron	11 mg/day (males 13–18 yrs) 15 mg/day (females 13–18 yrs)	Ferritin ≥ 30 µg/L = adequate for adolescents ≥ 15	[27,28,29]
		Screen athletes with fatigue or low endurance	
Calcium	1300 mg/day (14–18 yrs)	Include dietary sources in meals/snacks; dairy and fortified plant-based options recommended	[30,31]
Vitamin D	600–800 IU/day (15–20 µg)	Serum 25(OH)D ≥ 30 ng/mL optimal; supplementation may be needed	[30,32,33,34]

**Table 2:** Ferritin Thresholds by Age Group.

Age Group	Ferritin Threshold	Interpretation
Children (6–12 years)	<15 µg/L	Depleted iron stores
Adolescents (12–15 years)	<20 µg/L	Depleted iron stores
Athletes ≥ 15 years	<15 µg/L	Depleted iron stores
Athletes ≥ 15 years	15–30 µg/L	Low iron reserves
Athletes ≥ 15 years	≥30 µg/L	Adequate iron stores (clinical cut-off)

**Table 3:** Summary of supplements and ergogenic aid recommendations for adolescent athletes.

Supplement	Recommendations	
Protein Supplements	Use only when whole food intake is insufficient; choose third-party tested products	[18]
Creatine	May be considered for athletes in structured training under supervision	[85]
Omega-3 (EPA/DHA) supplements	May support recovery, reduce inflammation, and protect brain health	[64,66]
Vitamin D Supplements	May be needed due to low sun exposure and limited dietary sources; monitor serum levels for adequacy	[33,34]
Iron Supplements	60–120 mg elemental iron/day (if deficient)	[27,78]
	Take in morning with vitamin C; avoid with coffee or meals	
Energy Drinks	Not recommended	[86,87]
	Risk of excessive caffeine, sleep disturbance, and cardiovascular effects	

**Table 4:** Resources for locating qualified sports dietitians.

Country	Directory Name	Website/Resource
United States	Academy of Nutrition and Dietetics	eatright.org
Canada	Dietitians of Canada	dietitians.ca
United Kingdom	British Dietetic Association (BDA)	bda.uk.com
Australia	Dietitians Australia	dietitiansaustralia.org.au
Europe	European Federation of the Associations of Dietitians (EFAD)	efad.org
